# Post-Intensive Care Syndrome: A Problem Cannot Be Ignored in ICU. Comment on Inoue et al. Prevalence and Long-Term Prognosis of Post-Intensive Care Syndrome after Sepsis: A Single-Center Prospective Observational Study. *J. Clin. Med.* 2022, *11*, 5257

**DOI:** 10.3390/jcm12010268

**Published:** 2022-12-29

**Authors:** Jiayuan Ni, Yuan Ding, Liying Miao, Bin Zhu

**Affiliations:** 1 Department of Critical Care Medicine, The Third Affiliated Hospital of Soochow University, Changzhou 213000, China; 2 Department of Nephrology, The Third Affiliated Hospital of Soochow University, Changzhou 213000, China

Recently, we read with great interest the article by Inoue et al. [1], in which the authors found that the incidence rate of post-intensive care syndrome (PICS) at 3, 6, and 12 months after recovering from sepsis were 70%, 60%, and 35%, respectively. In addition, sepsis survivors who had PICS showed a lower 2 year survival rate. Sepsis is recognized as a global public health problem and a highly lethal disease in ICU patients. In recent years, with the improvement of anti-infective therapy and organ function support technology, the short-term survival rate from sepsis has been improved, but the long-term prognosis is still affected by physical, cognition, and mental impairment after ICU stay [2]. The physiological damages caused by sepsis have been widely concerning, but the cognitive and psychological disorders are usually neglected. The study has shown that the prevalence of moderate to severe cognitive impairment increased 10.6% among patients who survived from severe sepsis [3]. Meanwhile, sepsis survivors were prone to mental health problems such as anxiety, depression, and post-traumatic stress disorder (PTSD), which will increase the mortality of patients [4]. It is therefore likely that the high incidence of PICS would affect the treatment and prognosis of patients with sepsis. Therefore, the reasons for cognitive and psychological PICS in patients with sepsis are worthy of further elucidation.

Sepsis-associated encephalopathy (SAE) is a diffuse brain dysfunction secondary to systemic infection and mainly manifests as cognitive impairments and psychiatric diseases. SAE is initially considered as a fully reversible complication during sepsis. However, some scholars have found that the pathophysiological processes related to SAE, such as blood-brain barrier dysfunction, cerebral blood flow impairment, neural inflammation, and glial cell activation, may lead to permanent neurocognitive dysfunction and functional impairments [5]. In addition, the correlation between sepsis-associated delirium and long-term cognitive dysfunction has received widespread attention. Sepsis-associated delirium is defined as a manifestation of brain organ dysfunction caused by infection, accompanied by changes or fluctuations in mental status, confusion, or altered level of consciousness [6]. The occurrence of delirium is frequent in ICU patients with sepsis, and the duration of delirium is an independent predictor of long-term cognitive impairment [7].

The manifestations of mental health impairment in septic patients after discharge are various. Depression, anxiety, and PTSD are the most common psychiatric disorders related to PICS and are referred to as psychological PICS. Firstly, the prevalence of anxiety in ICU survivors is high and negatively impacts the prognosis of survivors. It has been reported that one-third of ICU patients experience persistent anxiety symptoms during their first year of recovery. Psychiatric symptoms during hospitalization and the occurrence of delirium were associated with post-ICU anxiety [8]. Furthermore, depression is also common in ICU survivors. Rabiee et al. [9] found that depression occurred in 29% of ICU survivors, and depressive symptoms could persistent through 12 months. PTSD during the post-ICU period is another common psychiatric disorder that can persist for a long time. PTSD symptoms occurred in 20% of ICU survivors at 1-year follow-up [10]. In this regard, sepsis is an independent risk factor of PTSD after critical illness [11].

In conclusion, PICS, characterized by cognitive and psychiatric disorders, is common in ICU septic patients. It not only aggravates the short-term mortality of patients, but also leads to a reduction in their long-term quality of life and ability to return to work. Therefore, we need to pay more attentions to neuropsychiatric disorders in ICU patients.
